# 

**Published:** 1892-08

**Authors:** 


### CONTENTS:

ORIGINAL ARTICLES: PAGE

Recent Investigations on Tetanus. By Simon J. J. Harger, V.M.D .............. 453
Notes on Parasites. By C. W. Stiles, Ph. D .............. 464
The Etiology of Southern Cattle Plague—Texas Fever. By Frank S. Billings... 467
"So-called" Spinal Menigitis. By A. W. Clement, D.V.S .............. 486

EDITORIAL:

The Meeting of the United States Veterinary Medical Association .............. 489
Veterinarians as Educators .............. 490
Importation of Horses .............. 491

SELECTIONS:

Fibrous Ankylosis. By J. A. Nunn, D.S.O., F.R.C.V.S .............. 496

Malpositions of the Colon. By Thomas Walley, M.R.C.V.S .............. 499

The Staining of Tubercle Bacilli in Milk. By J. McFadyean, M.B., B.Sc .............. 503

COMMUNICATIONS:

Contrastive Coloring in Horses. By Horace J. Smith .............. 507
Correction of a Clipping. Williamson Bryden .............. 509

SOCIETY PROCEEDINGS:

California State Veterinary Medical Association .............. 510
Massachusetts Veterinary Association .............. 513
The Indiana Veterinary Association .............. 515

